# Using Complexity and Network Concepts to Inform Healthcare Knowledge Translation

**DOI:** 10.15171/ijhpm.2017.79

**Published:** 2017-07-10

**Authors:** Alison Kitson, Alan Brook, Gill Harvey, Zoe Jordan, Rhianon Marshall, Rebekah O’Shea, David Wilson

**Affiliations:** ^1^Adelaide Nursing School, Faculty of Health and Medical Sciences, University of Adelaide, Adelaide, SA, Australia.; ^2^Green Templeton College, University of Oxford, Oxford, UK.; ^3^Adelaide Dental School, Faculty of Health and Medical Sciences, University of Adelaide, Adelaide, SA, Australia.; ^4^Institute of Dentistry, Queen Mary University of London, London, UK.; ^5^Alliance Manchester Business School, University of Manchester, Manchester, UK.; ^6^Faculty of Health and Medical Sciences, The Joanna Briggs Institute, University of Adelaide, Adelaide, SA, Australia.; ^7^Adelaide Medical School, Faculty of Health and Medical Sciences, University of Adelaide, Adelaide, SA, Australia.

**Keywords:** Knowledge Translation (KT), Evidence-Based Practice, Implementation Science, Complex Adaptive Systems (CASs), Complexity, Networks, Integrated Knowledge Translation

## Abstract

Many representations of the movement of healthcare knowledge through society exist, and multiple models for the translation of evidence into policy and practice have been articulated. Most are linear or cyclical and very few come close to reflecting the dense and intricate relationships, systems and politics of organizations and the processes required to enact sustainable improvements. We illustrate how using complexity and network concepts can better inform knowledge translation (KT) and argue that changing the way we think and talk about KT could enhance the creation and movement of knowledge throughout those systems needing to develop and utilise it. From our theoretical refinement, we propose that KT is a complex network composed of five interdependent sub-networks, or clusters, of key processes (problem identification [PI], knowledge creation [KC], knowledge synthesis [KS], implementation [I], and evaluation [E]) that interact dynamically in different ways at different times across one or more sectors (community; health; government; education; research for example). We call this the KT Complexity Network, defined as a network that optimises the effective, appropriate and timely creation and movement of knowledge to those who need it in order to improve what they do. Activation within and throughout any one of these processes and systems depends upon the agents promoting the change, successfully working across and between multiple systems and clusters. The case is presented for moving to a way of thinking about KT using complexity and network concepts. This extends the thinking that is developing around integrated KT approaches. There are a number of policy and practice implications that need to be considered in light of this shift in thinking.

## Background

A 1998 landmark study reviewing the quality of care in the United States indicated that some 30% to 50% of care delivery was not in line with best available evidence.^1^ Over the ensuing 18 years, we have witnessed a growing policy focus on the translation of research-based knowledge into routine healthcare. In June 2000, the Clinical Research Roundtable was convened by the (US) Institute of Medicine to address the concern that scientific research was not translating into tangible human benefit.^2^ Similar discussions regarding research translation were taking place in other international health systems, with a general consensus that there were at least two major obstacles: (1) the translation of basic scientific discoveries to clinical application; and (2) the translation of clinical research into routine healthcare practice and decision-making. The complexity of the challenge ahead was acknowledged from the early days of discussion: “it has become clear that these 2 translational blocks can be removed only by the collaborative efforts of multiple system stakeholders” (p. 1278).^2^

Some 16 years on from the Clinical Research Roundtable, translation remains a difficult and enduring problem for health systems. The CareTrack study in Australia, based on similar methods as the US research,^1^ came to almost the same conclusion, that Australian patients only received care judged to be appropriate (that is, in line with evidence-based guidelines) 57% of the time.^3^ Despite knowing this, there has not been a collective push or an articulated conceptualisation of knowledge translation (KT) that encompasses all sectors: research, education, clinical, community and government (including funding and policy), within which to work. Nor has there been a KT framework that brings together key areas of activity required for translation in collaboration with the sectors, despite recognition in certain other areas such as innovation studies^4^ of the need to generate greater synergy and collaboration. It would seem that the provenance of the KT discourse within health is mediated more through step-wise progressions rather than an acknowledgement of the need for synergy and experimentation.

It is against this backdrop that we put forward the central argument developed in this paper; that in order to progress the science and practice of KT in healthcare, we need to re-conceptualize the way we think and talk about translation.

Historically, models representing the KT process have tended to depict it as a pipeline that moves from knowledge generation through a process of synthesis (for example, in the form of systematic reviews and clinical guidelines) to uptake and implementation in practice.^5^ This is premised on an assumption that producers and users of research are two separate groups or communities and that translation occurs in a rational, linear way to move knowledge from producers to users.^6^ When KT appears to be slow or incomplete, the metaphor of ‘translational gaps’ is used and various bridging strategies such as the use of research navigators and knowledge brokers are proposed to try to close the gap. Other conceptualisations extend the ‘pipeline’ metaphor to represent it as a cyclical process where the representation is of knowledge being used within a process of planned change.^7^

KT research repeatedly highlights the complexity of the process and the multiple factors that determine whether and how research-based knowledge finds its way into healthcare policy and practice.^8,9^ Factors include the negotiated and contested nature of evidence in healthcare decision-making, such that good research is not sufficient to ensure its uptake in practice.^10-12^

Greenhalgh and Wieringa^13^ have identified that the use of current KT models and metaphors may inadvertently close our minds to alternative framings and analysis of this complex field; it is noteworthy that there have been few alternative conceptualisations put forward to help explain how we might approach this challenge by lateral thinking.^14,15^ Innovation studies have attempted to move beyond linear thinking with variable success, for example, with the chain-link model^4^; health is still exploring alternatives within confined systems.

Given the growing acknowledgement of the inherent complexity in KT, it seems appropriate to explore KT in multi-dimensional, iterative and flexible ways drawing on approaches used in the social sciences and industrial organizations.^6,16^ This includes recognising the important role of actors, relationships and networks, in order to actively mobilize knowledge between the stakeholder groups involved,^17^ and embracing collaborative processes of knowledge production and use.^18^

The use of complexity to explain what happens in health systems is growing.^19-22^ Graham and colleagues have always been strong advocates of taking an integrated approach to KT which requires the integration of KT principles into every step of the research process.^7,23^ Kitson et al,^24^ similarly developed a method to co-create a KT approach within a population health study. The trend therefore is toward more integrated and dynamic representations of KT.

The ideas presented in this paper have been developed and refined by a cross-faculty interdisciplinary team within an academic health and medical science faculty. The primary goal is to put forward a way of thinking about KT using complexity and network concepts and principles which extend the thinking around KT.

Complexity concepts have been utilised in both healthcare and educational literature to facilitate understanding about the emergent nature of both the contexts and the participants within them.^22,25-28^ Also, within the KT literature there has been a growing number of studies that have looked at particular elements of KT using aspects of complexity thinking.^20,29-31^ For example, a recent scoping review investigated whether planning with complexity in mind was effective in evaluating the effectiveness of an intervention.^32^ However, these discussions have not considered nor been able to demonstrate how complexity would look or apply in KT more broadly. They tend to miss the *holistic*, *dynamic and convergent interactions* of KT, working at the interface of multiple systems in ways that are often unpredictable. Specifically, we propose the application of complexity and network principles to embrace the dynamic and interactive nature of KT in the real world in order to provide more sustainable KT.

### Approach

The response to the ‘wicked’ problem was to generate a strategic framework effective for KT across an interdisciplinary Faculty oh Health and Medical Sciences (FHMS) in one research-intensive university in Australia. The purpose was to improve the shared understanding of research contributions; increase interaction and work collaboratively with key stakeholders to optimise the positive impact of the research activity. Our cross-Faculty group identified that academic colleagues (on the spectrum from bench to clinical, applied and health service researchers as well as academics with more of a learning and teaching emphasis) conceptualized KT as either a pipeline or a cyclical model or as an interactive, complex process.^33^

This preliminary mapping work, along with previous experience in the KT space^8,24,33-36^ encouraged us to conceptualize KT as a multidimensional, dynamic, complex, integrated process. The following inductive approaches were used to develop the KT Complexity Network model:

**Table 1:** Descriptions of KT Research, as Conceptualized by Different International Research and Working Groups

**What**	**Who**	**Why**
*Roadblocks in the translational research pipeline* T1 Roadblock 1 was the ‘transfer of new understandings of disease mechanisms gained in the laboratory into the development of new methods for diagnosis, therapy, and the prevention of their first testing in humans’ (p. 1279). T2 Roadblock 2 was the ‘translation of results from clinical studies into everyday clinical practice and health decision-making’ (p. 1279).	Sung et al,^2^ 2003	Concern by researchers that the flow of research from basic to clinical research was held up at two key intersection points. First time that significant policy attention was focused on the ‘gaps’ or ‘roadblocks.’
*Translational research needs to embrace community networks* Community physicians need greater involvement in the translational research space. More practice based research involving community networks in trials and implementation studies was needed. A third gap – T3 – was identified, this being the gap between the clinical trial and implementation.	Westall et al,^37^ 2007	Concern that significant investment funds had gone into improving basic and clinical research infrastructure but little had gone into the more ‘applied’ end of research – either health service research or practice based research.
*Translational research needs to embrace HSR* Concern that little attention (and funding) was directed to HSR translational activity that translates research into practice. An additional ‘gap’ was articulated – ‘closing the gap and improving quality by improving access, reorganising and coordinating systems of care, helping clinicians and patients to change behaviours and make more informed choices, providing reminders and point-of-care decision support tools and strengthening the patient-clinician relationship’ (p. 211). Reinforcing the need to close the T3 gap.	Woolf,^38^ 2008	Concern by HSR community that little attention or funding was being directed at the application and testing of new knowledge into clinical practice.
*Translational research needs to embrace epidemiology and population health* Concern that the population level understanding of KT had been overlooked and a case was made for the addition of two extra ‘gaps.’ T4 was the gap between getting the knowledge from the practitioner to the patient and the wider population and the fifth gap, which was called T0, was the gap between population health and disease burden to scientific discovery research.	Khoury et al, 2010^39^	Concern by population health community that translational research policy and funding priorities had omitted their contribution to KT.
*KT activity can be divided into two distinct approaches: end-of-grant KT and integrated KT* End-of-grant KT requires researchers to develop and implement a plan for making potential users of the research aware of it. Integrated KT involves potential users in a more meaningful engagement throughout the research process. Integrated KT is about ‘collaborative, action oriented, participatory research and involves two-way interactions between researchers and knowledge users (clinicians, health systems administrators, managers, policy-makers, patients and the public’ (p. 2149).	Graham and Tetroe,^7^ 2008	Making the case for a more integrative approach to KT that places responsibility on the researchers to consciously engage knowledge users in the uptake of that knowledge. This can either be in a more traditional end-of-grant way or by creating a more dynamic partnership. The concept transcends the research differentiations and thus helps overcome the notion of ‘gaps’ or ‘roadblocks.’

1. Using the Institute of Medicine landmark publication^2^ as our starting point, we traced the development of the concepts around KT outlining the main contributions to the development and refinement of ideas (See Table 1).

2. Our understanding of KT moved away from referring to translation ‘gaps’ which had been identified in existing models, towards conceptualising the translation ‘space’ as ‘synapses of interaction and connectivity’ between elements of translation.

3. Once we had made this shift in our own thinking, we took this understanding and focussed on the general implicit and explicit assumptions of current models including the concept that often ‘users’ of knowledge were portrayed as peripheral and passive to KT, the emphasis being on the knowledge ‘producers’ and how they could move their knowledge to users; that ‘push-pull’ activity was sufficient to realise KT; that ‘bridging’ so-called gaps would greatly enhance the use of evidence in health practice; and the implicit assumption that multiple dimensions and multiple systems may be required to support KT but without building them explicitly into current models.

4. From this review, five key areas of the KT process (widely accepted as key aspects of planned action models) were identified (problem identification [PI], knowledge creation [KC], knowledge synthesis [KS], implementation [I], evaluation [E]). Current ways of conceptualising these KT process are limited by their linearity and boundaries; they need to be viewed as dynamic, unpredictable interactions that embrace constant adaptation in order to adopt new innovations and sustain new practices.

5. We then identified literature from other fields in which complexity has been considered and used this to develop different ways of thinking about KT.

In addition to our analysis of the literature, we also ran three interactive workshops with academic and research staff across the faculty over the period between October 2013 and June 2014. From these workshops:

1. We confirmed the five key areas of process in the KT Complexity Network model.

2. We generated and refined ways of conceptualising KT that reflected its integrated, dynamic and complex nature rather than being seen as a ‘pipeline’ or a cycle that had some predictable sequencing.

**Figure 1:**
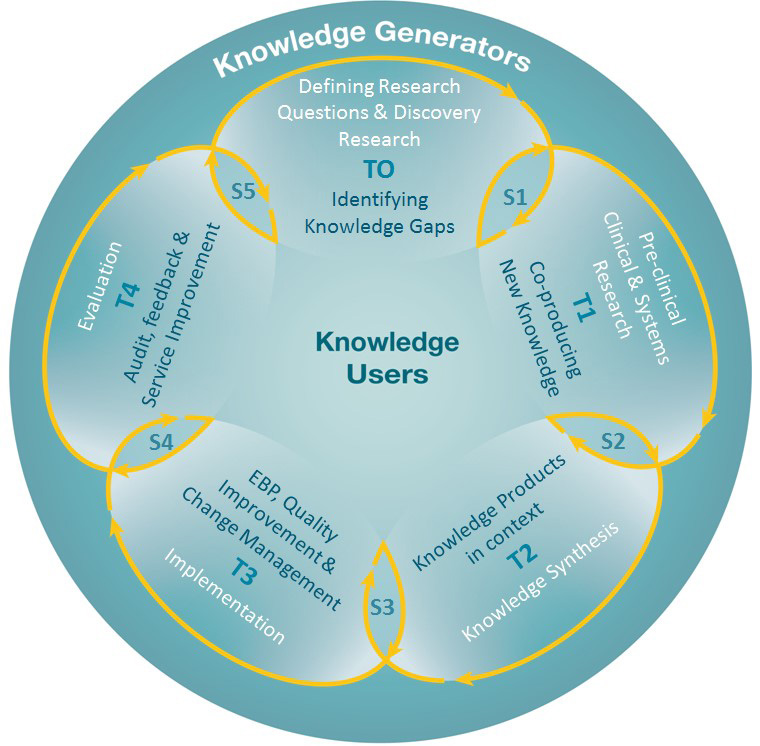
Initial Conceptualisation of Knowledge Translation (KT)Incorporating Gaps and “Synapses of Interaction and Connectivity.”

These steps generated an initial version of the model (see Figure 1).

**Figure 2:**
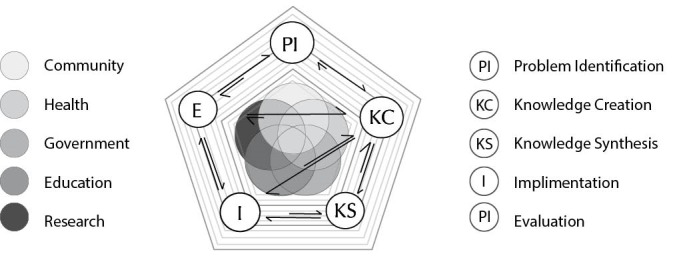
Refined Model of the Knowledge Translation (KT) Complexity Network Incorporating Sectors and Processes.

3. We then explored how colleagues thought about the main sectors with which they had to engage across the KT processes, namely, governments; the community including industry; and other complex systems. At this stage, we realised the model was too static; it did not sufficiently display the dynamic interactions and complexity we had come to recognise as important.

4. As a result, we refined the model through application of complexity literature and the experience of our team members (in particular DW and AHB) (Figure 2). This consolidated our thinking in order to provide a theoretical and conceptual framework which could help colleagues understand how they may be able to translate knowledge more effectively.

5. To ‘test’ our concept, we retrospectively mapped our model to projects which had documented KT outcomes: increasing embryo quality; and forensic age determination based on dentition (full detail published separately).^40^

6. Following this retrospective mapping exercise we further refined the model to its current form. This was only possible after further interrogation of complexity and networking literature.

The following section will elucidate the nature of complexity and networking that lends itself to KT.

### Main Argument

**Figure 3:**
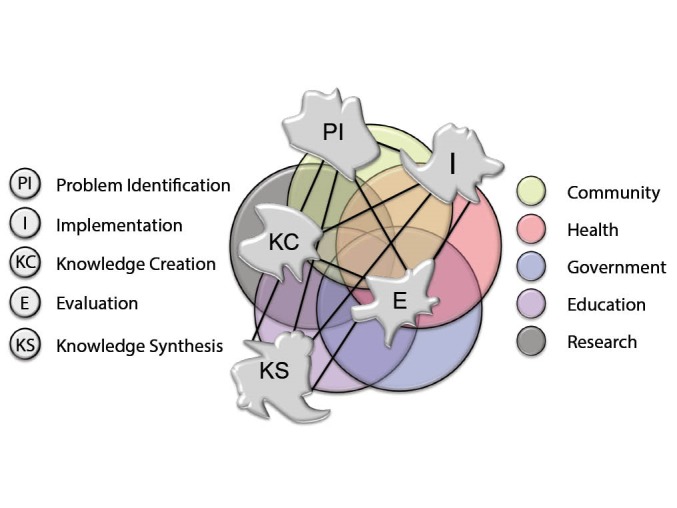
Current Knowledge Translation (KT) Complexity Network Model.

The current KT Complexity Network model (Figure 3) was produced to identify the core building blocks of any KT framework that had the job of creating and translating knowledge. Unlike other representations, the KT Complexity Network model does not represent this movement in a linear or cyclical way; it suggests that the direction of travel is dependent upon the decisions and actions of individuals and teams who can connect into, across and between multiple networks in order to achieve a desired outcome (adaption or acceptance of new knowledge). We argue that taking account of, and leveraging, these interconnections may build a more adaptive and sustainable approach to KT.

Central to this KT model are five sectors which can also be seen as complex adaptive systems (CASs); Research, Education, Health, Government, Community (including Industry). The five irregular-shaped clusters of the network; PI, KC, KS, I, E, function dynamically in space and time in the realm of CASs. Each sector and cluster may be weighted differently and interact more or less frequently (as indicated by the presence/length of the lines) depending on the needs of a given KT goal.

Before we introduce the concepts of complexity, consider the scenario in Box 1. We shall refer back to this scenario after we explain the concepts of complexity and networking as they shed light on the emergent KT Complexity Network model.

**Box 1. A Scenario Illustrating Successful Knowledge Translation**

A Tsunami has devastated an Asian country, leaving many locals and travellers deceased or homeless. The victims of the disaster need to be identified.

A forensic odontologist, acting as a local disaster response volunteer, decides to seek advice from an international colleague. The colleague connects with clinicians and researchers around the globe, across disciplines outside of her usual dental field. The group evaluates the existing evidence base; it is evident that existing methods and knowledge are limited and not capable of providing adequate solutions, particularly for identifying young children. The group embark on a project to create new knowledge, and synthesize that knowledge into a tool to aid human age determination by dentition.

As new knowledge is created, the individuals collaborate to evaluate the knowledge and the developing tool. They interact repeatedly with organizations and professional associations spanning diverse sectors, such as healthcare, government, research, education and community to further refine the knowledge. They establish a world copyright on the tool so that the tool can be made freely available for use around the globe and they disseminate their research through professional publications. The tool is validated through interconnection with the education and research sectors. The translated knowledge is available as electronic applications for the internet and mobile telephone, and is provided in multiple languages. This tool is used to teach students across several disciplines, and is also used to determine human age by dentition in areas removed from the original impetus of natural disasters. It provides a more accurate estimation of age than any prior tools and can be used for people from different ethnic backgrounds.

### Building Blocks for Knowledge Translation Using Complexity Principles

**Figure 4:**
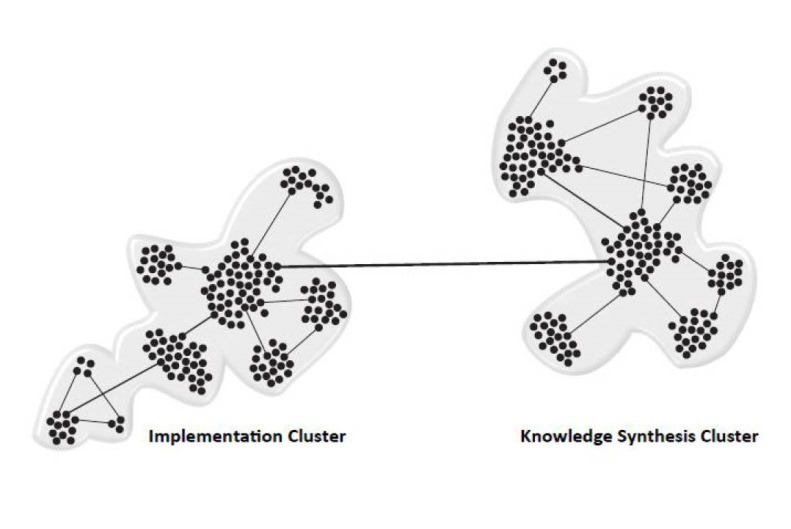
Representation of Two Clusters (Sub-networks) Within a KT Complexity Network. Each dot represents a node, while the lines are the main interconnections. The nodes at each end of the lines act as hubs and the thicker lines represent interactions between larger groups. The thickest line interconnects the two clusters. Abbreviation: KT, Knowledge Translation.

In the domain of complexity, network models (derived from network theory^41^) provide ways of understanding connectedness and the proximity of interactions much like stylised underground transport maps do for commuters. A structured network consists of nodes, hubs, and clusters (sub-networks) that facilitate multi-tasking.^42^ These terms are the ‘building blocks’ of complexity and network concepts and are defined in Table 2 and illustrated in Figure 4.

**Table 2:** Nomenclature Used and Working Definitions of the KT Complexity Network Elements

**Term**	**Explanation**
Node	A single agent (individual, process or virtual system) that interacts with other single agents (nodes).
Hub	A single agent that interacts more extensively with other nodes and becomes the champion for collective actions, within and between clusters.
Cluster	A sub-network made up of nodes and hubs. The sub-network comprises a number of nodes, some of which act as hubs, pursuing the same goals. A cluster may be a sub-network involved with key areas of activity (such as PI) or a sub-network within a sector (such as a university health science research group).
Network	A collection of nodes, hubs, clusters and the connections between them.
PI	The process by which societal challenges, issues or problems are formulated, defined and constructed to proceed to systematic investigation.
KC	Describes what is traditionally termed basic, clinical, pre-clinical, epidemiological, health services, and population health research approaches to answering health related problems.
KS	The rigorous and systematic generation of evidence-based products (patents, materials, tools, programs, and guidelines) for application in policy and practice.
I	The rigorous application of new knowledge into policy and practice in a theory informed and reflective way.
E	The explicit and systematic review of key processes of KT and broader objectives within and across a range of complex and interconnected sectors and networks.
CAS	Complex systems (eg, within Research Institutions, health systems) and KT processes (eg, PI, KC) that are a collection of diverse connected nodes or parts with interdependent actions. The behaviour of a CAS is generated by the adaptive interactions of its components.
KT Complexity Network	The umbrella term that describes the components of the overall network that connect and interplay in order for KT to occur. Different stakeholders collaborate within a dynamic discursive space to ensure that appropriate information is being developed, refined, and mobilised throughout the network to the appropriate nodes, hubs, clusters and sectors.

Abbreviations: PI, problem identification; KC, knowledge creation; KS, knowledge synthesis; I, implementation; E, evaluation; CAS, complex adaptive system; KT, knowledge translation.

We recognised that both actions and sectors are required to realise KT, and that these can be depicted as clusters in a network model. The sectors (research, education, health, government, community) provide the structures and systems to create, mobilise, teach or fund KT. The five interdependent clusters are key areas of process that are required for a truly integrated KT approach: PI, KC, KS, I, and E (see Table 2). These evolved from a synthesis of the models commonly used to represent KT.^37,39^ Whilst our five key areas of process are implicit in existing KT representations or models, we have made them explicit and also considered the dynamic nature of KT, which has not previously been captured. This dynamism reflects the interconnections between nodes, hubs and clusters which is more likely to facilitate adaptive and sustainable KT.

Texts about complexity^43-45^ emphasise investigating relationships between and among individuals, organizations and or systems and the resulting behaviours and outcomes.^46^ Within complex systems, these outcomes are often unpredictable, non-linear and emergent. One key characteristic of complex systems is the notion of ‘self-organization.’^47^ This is described as “the process by which agents in a system interact with each other according to their local rules of behaviour without any overall blueprint telling them what they are to accomplish or how to do it” (p. 290).^48^ A Complex Adaptive System is a collection of diverse parts interconnected such that the organization (or organism) grows over time without centralised control (p. 43).^49^ The behaviour of a CAS is generated by the adaptive interactions of its components (nodes, hubs and clusters). Table 3 summarizes the main characteristics of a CAS, as utilized in this work.

**Table 3:** The Main Characteristics of CASs

**Characteristic**	**Description**
Agents/nodes (or hubs if they are leaders in a system)	Individuals, people, processes, or virtual systems and how information is exchanged. Agents respond according to their own capacity within various organizations. Control parameters include: rate of information flow, degree of diversity, richness of connectivity, level of anxiety and degree of power differentials.
Interconnections	The number and strength of connections within a CAS and interdependence has an impact that is influenced by relationship quality. Overall cooperation tends to be unsustainable when the group size exceeds a critical threshold.
Self-organization	The activity of the agents, the system’s individual components and the system itself, as it moves from seemingly disorganised and random to highly differentiated and interdependent. Once ‘local rules’ have been established and ‘bottom up’ adjustments have occurred, this pattern of ‘causal circularity’ serves to stabilise the CAS. Shared ‘meaning’ is created and this becomes part of the CAS as ‘organizational memory.’
Non-linearity	The non-predictable nature of the relationships, behaviours and interactions that are created and occur within CAS. It also refers to the fact that small changes in inputs, physical interactions or stimuli can cause large effects or very significant changes in outputs.
Emergence	This is a macro-level occurrence that results from local-level interactions where the agents constantly act and react to the behaviour of other agents involved in the activity. Such interactions are considered to be random and may form new paths/shortcuts, create new phenomena in the system or even maintain ‘roadblocks’ and the status quo.
Dynamics	Dynamical Systems Theory (or Dynamics) concerns the description and prediction of systems that exhibit complex changing behaviour at the macroscopic level, emerging from the collective actions of many interacting components.
Co-evolution	CASs exist within an environment, but they are also part of their environment. Current and future behaviour of a CAS is linked to its history and environment. Over time the environment changes, and in turn, the CAS needs to change to ensure best fit. Each change causes the need to change again, and so it goes on as a constant process. Similarly, one CAS can interact with others and as a result, will change.

Abbreviation: CAS, complex adaptive system. Sources: Eidelson^49^; Miller and Page^50^; Mitchell^51^

It is important to understand that the inherent nature of a CAS directly influences efforts to translate knowledge. For example, while a translational project may consider a specific goal at inception, the unpredictable, emergent and self-organizing nature of complex systems means a variation of outcomes may actually be realised. The primary purpose of the CAS is not likely to be KT.

The five key areas of process or clusters can be thought of as sub-networks or even CASs in their own right. In reality, each cluster of activity represents the often siloed groups, based on focussed areas of specialty, that are already in place within the complexity of a KT network. Typically, each cluster evolves from a node or a series of nodes that are championed by a hub, an individual or individuals with a vision to move a specific initiative forward. Often individual hubs, nodes and clusters are very productive but may lose sight of the ultimate goal of KT. It may be that KT is not necessarily the primary purpose of the existing sub-network. This siloed approach must be overcome in order to extend the cluster activity through the entire KT Complexity Network.

Healthcare organizations, academic institutions, research facilities, government organizations and the broader community are all important sectors with which the five key clusters of KT processes need to engage. They can also be perceived as CASs, generating their own networks of nodes, hubs, and clusters as they pursue their primary tasks. To investigate this Brook et al^40^ retrospectively analysed how researchers had collaborated with national and international colleagues and with the key institutions and governments following the 2004 Indian Ocean Tsunami (the scenario in Box 1). This demonstrated how different ways of understanding and engaging with the problem (in this case using dentition to assist in identifying the age of the younger victims of the tragedy) were required. As a result of this new way of thinking and engaging with the problem, a highly relevant solution (a dental atlas) was generated which has now become the accepted standard.^52^ This retrospective analysis is adapted and summarise in Box 2, to illustrate the concepts of complexity and networks.

Complex Adaptive Systems encompass ideas, concepts and tools that can be applied across multiple disciplines. They demonstrate the property of emergence; where macro-level properties arise from the interactions of lower level activities.^50,53^ They are robust, and within them, cumulative small occurrences have the ability to suddenly pass a critical threshold and produce large events.^44,53^

How knowledge is created and mobilised within social CASs is determined by the relationships and shared understandings of what the benefits and incentives are for the movement of that knowledge. Understanding of such benefits may be explicit (as in the form of a set of objectives, mission statement or goals such as shown in development of the dental atlas) but more often they are implicit, reflecting the common consciousness or prevailing motives, values and relationships of a group of colleagues, a team or a network who work together to create a common goal. This is how the human age determination project started: to identify victims of a disaster.

Effective KC, movement and mobilisation will depend on how nodes and hubs can successfully interact within and between clusters. Clusters will have their own methodologies for generating and refining knowledge as well as preferred approaches for communicating it within and between clusters. The ‘boundaries’ (in traditional KT models called ‘gaps’) around clusters are dynamic: they can adapt and adopt according to the activities within and between the clusters. As Box 2 illustrates, this dynamic interconnectivity was crucial for identifying problems, creating knowledge, and synthesizing that knowledge into a tool to enable accurate dental age determination.

**Box 2. An Illustration of Complexity and Network Within the Tsunami Scenario**

A Tsunami has devastated an Asian country, leaving many locals (CAS – community locals) and travellers (CAS – community foreigners) deceased or homeless. The victims of the disaster need to be identified (PI important to community and government).

A forensic odontologist (a node within the health sector) acting as a local disaster response volunteer, decides to seek advice from an international colleague (health and researcher node). The colleague connects with clinicians and researchers around the globe (node becomes a hub and generates an international cluster), across disciplines outside of her usual dental field. The group evaluates the existing evidence base (repeated movement between PI, KC and E across health, community, research and government sectors); it is evident that existing methods and knowledge are limited and not capable of providing adequate solutions, particularly for identifying young children (E). The group embark on a project to create new knowledge (KC), and synthesize that knowledge into a tool to aid human age determination by dentition (KS).

**Figure 5:**
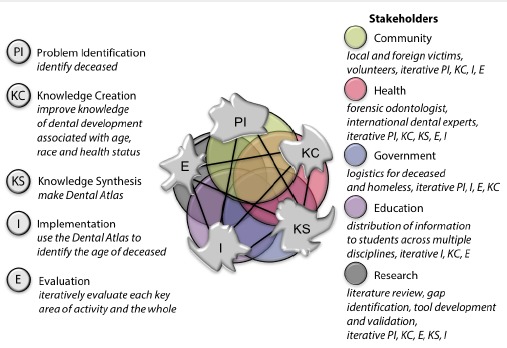


As new knowledge is created, the individuals collaborate to evaluate the knowledge and the developing tool (E and KS). They interact repeatedly with organizations and professional associations (reflecting dynamism, non-linearity, and self-organization) spanning diverse sectors, such as healthcare, government, research, education and community to further refine the knowledge (all examples of CASs which give rise to emergence and co-evolution). They establish a world copyright on the tool (KS) so that the tool can be made freely available for use (I) around the globe and they disseminate their research through professional publications (I). The tool is validated through interconnection with the education and research sectors (I, KC, and E).

The translated knowledge is available as electronic applications for the internet and mobile telephone, and is provided in multiple languages (I). This tool is now used to teach students across several disciplines (education), and is also to determine human age by dentition in areas removed from the original impetus of natural disasters (new networks and clusters within government and healthcare). It provides a more accurate estimation of age than any prior tools and can be used for people from different ethnic backgrounds. This is a real scenario, mapped to the KT Complexity Network Model, adapted from Brook et al.^40^

### The Knowledge Translation Complexity Network Model – Where Clusters Interconnect

The challenge remains around how the knowledge then mobilises across clusters; if we are proposing that the ‘space’ to be traversed is not characterised by ‘gaps’ but by ‘synapses of interaction and connectivity,’ then facilitating opportunities for early engagement of the sectors is vital in order for these connections to take place. The larger systems – whether located in community or government sectors or the clinical practice community (as illustrated in the scenario), must be engaged from the earliest stages. This highlights that the KT Complexity Network is not simply defined by the sum of its parts (nodes, hubs, clusters); relationship between these parts is of paramount importance. The main qualities of the KT Complexity Network is that it is a Complex Adaptive System comprised of its agents and characterised by interactions, self-organization, non-linearity, dynamics, emergence and co-evolution as defined in Table 3.^49,50^

Now that the theory has been introduced, consider the scenario again, in Box 2.

It is where the sector and activity clusters interconnect that the characteristics of CASs can be observed: agents will respond to information, creating multiple interconnections that move towards new understandings and insights. This co-evolution of new meaning enables knowledge to be created and move through clusters, and consequently, throughout the entire KT Complexity Network.

The embryo culture medium example (Box 3) illustrates the importance of timely interconnectivity and the impact that failure to engage important clusters (the clinical world) had on the overall success of the venture.

**Box 3. Illustration Providing a Demonstration of the KT Complexity Network**

**
Developing an Embryo Culture Medium to Improve Outcomes for Women Undergoing In Vitro Fertilization
**

PI began with the observation and personal experience of a bench researcher (a node within the research sector or KC cluster); that following in vitro fertilization (IVF), embryos were dying because the embryo culture medium was inadequate. This observation was shared with the researcher’s colleague (a node in both the KC and PI clusters, located within research and community), who was trying to conceive. In broader consultation with members (nodes) of the research team (the researcher has now created a hub or Knowledge Creation cluster) this concept was tested and found to have a sound scientific basis. The PI was refined which led to the creation of additional knowledge to improve in vitro survival of embryos. Once completed, further KC and E were undertaken (by the clusters connecting the research and healthcare sectors) in the form of large-scale clinical trials (involving community, government, and research). These proved positive and confirmed that the new medium was indeed superior to standard approaches. A patent was obtained for the created knowledge, from development of new connections beyond the KC cluster and research environment; this required contact with KS clusters in community (patent lawyers across global jurisdictions) and government.

However, there was a perception by many clinicians involved in implementation (I) of IVF that a new culture medium was not required and therefore, despite the new, superior evidence, clinicians were reluctant to change what they did. Existing commercial relationships with media suppliers and potentially commercial strategies by competing companies (industry representatives within community sectors) were additional likely factors in their reluctance to adopt the superior medium. Eventually research clinicians were willing to participate in one of the largest clinical trials of its kind to further test the effectiveness (KC, E) of the new medium. At this stage there was interaction of the KC, E, KS and to a limited extent, the Implementation clusters, and with four of the sectors: research, government, health and community.

**
What Was Learnt From it?
**

Although the KT moved reasonably quickly within PI and KC clusters due to the co-location of the originators of the knowledge, significant barriers were encountered at the KS and implementation stage (I). The pre-clinical, clinical trial and clinical application phases of KT were predominantly confined to the KC cluster, when this would ideally have involved broader interconnectivity with healthcare professionals and organizations outside the KC cluster. When later attempts were made to engage those clinicians and change clinical behaviours, challenges arose because engagement reflected an intermittent pattern (rather than ongoing, iterative manner), and there was some reluctance to acknowledge that a novel approach was required or beneficial (there was no coevolution). The patent holders (KS) on the process acknowledged they had limited interaction with patent agents, industry, clinicians and so when it became time to engage these groups in the process there were significant delays in arranging the interactions and developing the shared understanding of the goals of the KT. For example, the requirements of local government systems were different to those of international governments. The very nature of each of these diverse and separate, but interconnected, CASs slowed adaptation and evolution of the processes within the very systems which were needed to progress KT.

Engaging with Implementation (I) people (the clinicians) earlier in the process would mean that the significant attention focussed on KC and KS nodes would better prepare the KT group to succeed as they reached the later stages of the KT process. Additional interaction with community and those who were involved with PI may also have improved KT success, by providing advocates for change.

**
Take Home Message
**

Because this KT process was conceptualised traditionally as a pipeline, the activities within the self-organizing KC cluster retained the siloed approach, having limited interaction with other clusters until the process in that cluster was complete. Similarly, isolated interactions with the sectors such as healthcare organizations did not create a shared vision of all the KT stakeholders of other sectors and clusters. While the hub had formed sufficient connections to establish a network, the lack of accounting for local-level random and reactive behaviour (emergence), the complexity of numerous interconnections (dynamics) and the changing environment in response to those interactions (co-evolution) hampered KT progress. On reflection, the originating researcher (the hub now in the midst of the network) states that the process may have benefitted from earlier recognition of these interconnections and the unpredictability of such planned, interactive activity.

This interaction of cluster activity on many levels due to the interconnections, emergence and co-evolution is exemplified by the KT Complexity Network model; a fully developed KT Complexity Network is a meta-process that brings the building blocks together as represented statically in Figure 3. The interactions within and between the activity clusters and other CASs are not linear or circular but rather dynamically interactive. Figure 3 illustrates the dynamic nature with irregular boundaries around clusters of key process and interconnecting sub-networks. It is noteworthy that each cluster within the KT CAS and the other CASs may be weighted differently at any point in time to facilitate more or less frequent interaction, depending on the needs of a given KT goal. Engagement with each key activity cluster is not necessarily chronological in nature; interaction occurs regularly in anticipation of, and in response to, evolving environments of the clusters, individual CASs and the wider network. Dynamic expansion and contraction of interaction is key, as is a continuous evaluation of processes to permit evolution and provide a sustainable complex adaptive KT system. This representation of KT as a complex network means that knowledge movement can be traced but not necessarily mandated; the success will be dependent upon the hubs, nodes and cluster activity and their ability to connect with other clusters.

The interconnections within each cluster help accomplish specific tasks while the hubs coordinate communication between many functions. Thus both space and time are important and the connections between people and ideas influence outcomes. Simple interactions between neighbours can lead to complex group behaviours.^54^ Network thinking for organizations focuses on fostering the positive connections and relationships between individuals to produce novel outcomes and to make sense of situations, leading to concerted actions.

## Discussion

Making the key areas of KT process explicit helps engagement with other clusters throughout the entire KT Complexity Network. The movement within and between clusters depends on the energy and synergy driving the initiative; the number and location of potential hubs and nodes; the urgency of the problem; and the collaboration between multiple sectors and process clusters, to name but a few. The use of complexity and networks is a departure from conventional KT thinking, however sharing of this KT Complexity Network model in Complexity fields has been well received.^40^ This affirms its contribution to the complexity and network community; now the challenge is to explain how it might help in the healthcare policy, practice and research communities.

The different sectors, including government, community, health, education and research, have become increasingly concerned with KT, but present key performance indicators (KPIs) and funding mechanisms tend to reward working independently to identify solutions to common problems. This independent effort severely hampers progress. Equally, within the traditional KT way of thinking, different research siloes have been created and it is increasingly difficult to facilitate meaningful conversations between expert groups who, out of necessity, see the world in very precise and specialist ways. This disconnect has created multiple challenges in understanding the ways that KT needs to cross boundaries and promote better collaboration and shared understanding.

Policy initiatives that have attempted to overcome this fragmentation, such as the establishment of formal academic-health service partnerships to create the type of collaboration proposed by Sung and colleagues^2^ appear blinded to the issues of complexity. Several examples include Academic Health Science Centres and Networks (US and UK), Collaborations for Leadership in Applied Health Research and Care (UK) and Advanced Health Research and Translation Centres (Australia).^55^ They are premised on an expectation that bringing producers and users of research together in a formal collaborative relationship will help to ease the movement of knowledge within the overall health sector. However, evidence emerging from evaluation studies questions the assumption, indicating that the “policy of setting up translational networks is insufficient of itself to produce positive translational activity” (p. 192).^56^ The policy question then is how can we incentivise the clusters to engage more effectively? Will incentivisation work if we have created nodes, hubs and clusters that are dynamically connecting with other clusters throughout the KT Complexity Network?

It may be that the *structural solutions need to be underpinned by complexity and network thinking* so that the leaders within the systems understand they need to be looking for individuals (or nodes) who will act as ‘hubs,’ interacting with other nodes within and between clusters. Such modelling may indeed be evident from the early work on roles such as knowledge brokers^57^ and wider work relating to capacity building for knowledge mobilisation.^58^

To articulate the network and facilitate better outcomes, one needs effective connectivity between the five core areas of KT process and the organizational Complex Adaptive Systems. Translating knowledge can be improved by encouraging timely and dynamic interaction of each of the five clusters of activity to ensure there is alignment of each cluster’s activity with the common goal of KT. This may require removal of structures which limit the formation of the diverse interconnections required for the KT Complexity Network to function. Resistance to change might be due to the limitations that existing structures have on people’s ability or desire to develop and maintain these positive connections. An alternative, and perhaps easier way to cross these perceived boundaries, is through the co-location of nodes or hubs across several clusters and CASs. This would align with the concept of ‘boundary objects’ as discussed by researchers in innovation or organization science fields.^59,60^

Constant evaluation of the activity within and between sectors and clusters enables mapping of relationships, increasing adaptation and focussing effort and resources to particular systems or clusters to optimise success of the KT. This means that the flexible cluster boundaries and interactions between clusters in any KT activity will change over time and over the course of the KT activity. Mapping the dynamic change in relationships and events may in itself lead to better, more effective, precise ways of evaluating the effectiveness of the KT process.

This presents a new way of thinking and evaluating the key performance indicators (KPIs) for successful KT; to reward group effort and capacity to be an effective participant in the KT team. The trend to reward research activity by evaluating ‘impact’^61^ is another policy lever that may facilitate more interconnectivity with different groups working together. Taken to its logical consequence one could envisage ‘an evaluation of impact report cards’ covering all the KT clusters – how the problem was identified and who was involved; the quality of the KC activity; the plan around synthesising the knowledge and how the implementation and evaluation activities were activated. This would help to build up multiple case studies to inform future activity and contribute to the KT evidence base.

KT as conceptualised within a complexity frame, may occur in a variety of ways across a range of sectors with seeming disorder. Each KT scenario is expected to be different, involving different CASs at different times and requiring flexibility and perhaps quite different responses at each point in time. No one scenario represents a wrong or right approach, but flexibility is required to ensure that interactivity is generated from multiple sources as appropriate and needed. Equally, there may be many scenarios that overlay one another, either with regard to the same activity or multiple linked activities and these may be occurring across the same or different timespans. The more scenarios that overlay one another, the more complex the network of interactivity becomes. The net effect is a “web” of cross-disciplinary, multi-level, multi-sector platforms, through which knowledge must travel in order to add value to any of the CASs within which it is located. The KT Complexity Network model allows for this flexibility through its in-built recognition of complex networks.

Complexity adds to the challenge of tracking, anticipating, mapping and evaluating the impact of knowledge in systems. However, it is what we have to understand if we are going to improve the uptake of knowledge into policy and practice. For example, consider the challenge facing society of managing obesity and the multiple stakeholders required to work together to understand and to implement new approaches. Attempts so far have proven unsuccessful in managing the complexity of this challenge and it would be good to take this as one of the first ‘wicked problems’ that we expose to the KT Complexity Network model to begin to map knowledge mobilization.

The KT Complexity Network model could be used to identify and harness, explicitly, activities and interconnections, potentially resulting in more sustainable KT. To improve KT success with a pre-determined goal, inclusion of anticipatory planning responses, constant evaluation and engaging the best nodes could aid in managing the unpredictability. For example, much of the success of the National Innovation System (NIS) has been credited to the originators who are co-located across different sectors^60^ (our CASs), ideally placed to capitalise on the inherent unpredictability by implementing strategic initiatives, identifying opportunities, adapting delivery or driving dissemination or translation of knowledge.

By definition Complex Adaptive Systems are self-assembling and it will be the individuals or agents within the CAS who will determine whether the new information will lead to different interactivity, and produce and implement novel outcomes. *This is why it is vital to go back to the proposed primary purpose of the KT Complexity Network: it is to optimise the effective, appropriate and timely creation and movement of knowledge to those who need to know about it in order to improve what they do*. It is also important to note that by being more conscious/aware of the dynamic interactivity of the KT Complexity Network, new knowledge about KT has the potential to be created. *If individual agents (nodes), particularly those with a greater number of connections (hubs) lose sight of this, then they undermine the effectiveness of the KT Complexity Network to realise its optimum goal because their actions conform to and are determined by local priorities rather than the overall vision of moving knowledge to where it can be best used.* Within the complex systems, individuals emerge as hubs influencing actions that lead to knowledge mobilization.^54^

To optimise KT activity, those involved in any of the five core clusters of the KT process need to be able to move throughout and between the clusters in order to understand their contribution to the process. The example given in Box 3, describing the development of an embryo culture medium to improve outcomes for women undergoing IVF, illustrates what happens when due attention was not given to the implementation cluster in the early activity of the research plan. There are many more examples we could use that can illustrate both the successes and challenges in KT and such mapping will help to generate deeper understanding of how to optimise KC and mobilization in the future.

The practical consequence of this means that health professionals, researchers, clinicians, community members and policy-makers need to have ways of connecting with one another both virtually and in real time. These engagements may be spontaneous or facilitated by local champions to determine the next points of connectivity and interaction required to enable KT. Intuitively, this makes sense from an individual influencing and leadership perspective. Scaled up into trying to understand how multiple leaders influence knowledge movement across multiple sectors is more difficult to grasp – however, this is the challenge we now face and this is where the KT Complexity model is trying to help.

This way of thinking about KT already highlights a number of areas for policy and practice change. In viewing KT as an interactive and iterative process, it is clear that much more thought needs to be given to how we facilitate greater shared learning and interaction of agents across the KT network. Deliberate strategies to reward interdisciplinary collaboration, flexibility and partnerships with practice settings need to be developed and more thought needs to be given to academic success criteria so that impact of new knowledge is valued as much as a success as is patent application or a high citation journal paper.

Currently, less interest or attention is paid to the PI, I or E clusters; we propose the KT Complexity Network supports true consideration of these important activities. These areas need significant and sustained investment if we are to really optimise the impact KC has to our society. If this were to happen, our ‘problems’ would then be those of true community need, our stakeholders and professionals at the patient or community interface would be naturally motivated to support proper implementation, and, our service providers would be in a prime position to evaluate new pieces of knowledge or new methods of treatment and practice that are highly valued to them and interwoven with their current practice following implementation.

This has not been an easy journey for the Faculty of Health and Medical Sciences itself; to date there are still cynics who believe this is overcomplicating a process. However, when invited to reflect on their own challenges in translating knowledge into practice researchers have uncovered similar patterns and issues to those we identified. This generates new conversations and takes that monumental step towards understanding the KT Complexity Network model. The biggest challenge is to move away from the security of the linear-rational thinking into acknowledging that life is much more complex and unpredictable. It is only when people sit together and engage in these conversations that the true synergies emerge. Paradoxically, creativity and curiosity are the true innovators in science.

Next steps in the KT Complexity Network model refining process are to continue the conversation by seeking feedback from the wider KT community, including cross-sector representation, about how this way of thinking about KT can explain and inform our work to improve the movement of knowledge throughout systems. We will continue to collect KT examples and apply them against the propositions within the KT Complexity model. We are also generating a set of evaluative criteria that will help us understand how each of the clusters within the KT Complexity Network mature and develop. This work will help us begin to generate the guiding principles or ‘simple rules’ required for CASs to operate.

Viewing KT through a complexity lens enables an analytical approach to policy development and practice that is more likely to result in successful implementation and facilitate a co-evolutionary research approach that accounts for the complex needs of diverse stakeholders. However, it is challenging and very different from the way many of us have been trained to think as health professionals, researchers and policy-makers; education may remediate this. Using complexity may also improve understandings of the contexts where policy must be implemented and enable creation of constructive frameworks that ensure more cohesive and connected CASs for policy development and implementation.

To facilitate this, policy-makers, researchers, clinicians and other stakeholders need to recognise themselves as interactive, emergent and co-evolutionary agents in the KT Complexity Network and shape funding and investment strategies accordingly.

## Conclusion

We are proposing a different way of conceptualizing KT. Moving away from two-dimensional models that suggest a logical, predictable linear or cyclical representation of the movement of knowledge, we have used complexity and network principles to represent how KT could be conceptualised and operationalized. The emerging KT Complexity Network model emphasises the central importance of individual actors within networks and who interact with others, thus forming clusters of activity. This, we argue, is more likely to be achieved when KT acknowledges a network of Complex Adaptive Systems. The next task in this journey is to work collaboratively with stakeholders to generate the guiding principles or simple rules that normally reflect CASs. Such guiding principles will enable more widespread uptake and use of these ideas and facilitate others in applying the KT Complexity Network model. Ultimately, to accelerate KT it is incumbent that all stakeholders recognize and foster the dynamic, interactive nature of these complex systems. This is painstaking work, which will require individuals to interconnect with others outside their discipline and local working environment, like the FHMS team did to develop this emerging model. If we are to overcome the current perceived barriers of KT there are some assumptions, choices and considerations that, if we embrace and are aware of, may help to clarify the process and how it is manifested in policy and practice.

The success of the approach we are proposing requires us to reconceptualise KT as something that speaks to the decisions and motivation of individuals in the KT community as well as their collective and collaborative actions. If we are able to begin to think of KT in this interdependent, contingent, relationship centric way, we can then move to a position of understanding the implications of each CAS, and the decisions that are made within them, on the others throughout the KT Complexity Network. This shift is not simply one that requires minor adjustments to existing mental models: we are suggesting a transformation in the way we conceptualise KT in order to support more adaptive and sustainable translation.

**
To initiate the process of changing the way we think about KT, consider the following questions:
**

- How would you facilitate networks and supports nodes to flourish across Complex Adaptive Systems?
- How would you recognise and use the energy within systems rather than fight it?
- How do you know you have all the key stakeholders around the table at the same stage?
- How could the KT Complexity Network model have changed the outcomes of a previous piece of Knowledge in which you were involved in translating?

## Acknowledgements

We would like to thank the Translational Science Task Force for helping shape ideas and guiding our thinking, and the Faculty of Health and Medical Sciences for participation and support. We would also like to thank the extensive feedback provided by the reviewers; our article improved substantially from their input.

## Ethical issues

Not applicable, as no primary data was collected for this theoretical paper.

## Competing interests

Gill Harvey is on the Editorial Board for the *International Journal of Health Policy and Management*. The Faculty of Health Sciences, the University of Adelaide, Adelaide, SA, Australia provided research administration support for this work.

## Authors’ contributions

AK, AB, GH, ZJ, RM, RO, and DW were involved with initial conception and design or refinement of conceptual thinking; AK, AB, GH, ZJ, RM, RO, and DW were involved with drafting and critical revision of the manuscript; AK, AB, RM, RO, and DW provided administrative, technical, or material support.

## Authors’ affiliations

^1^Adelaide Nursing School, Faculty of Health and Medical Sciences, University of Adelaide, Adelaide, SA, Australia. ^2^Green Templeton College, University of Oxford, Oxford, UK. ^3^Adelaide Dental School, Faculty of Health and Medical Sciences, University of Adelaide, Adelaide, SA, Australia. ^4^Institute of Dentistry, Queen Mary University of London, London, UK. ^5^Alliance Manchester Business School, University of Manchester, Manchester, UK. ^6^Faculty of Health and Medical Sciences, The Joanna Briggs Institute, University of Adelaide, Adelaide, SA, Australia. ^7^Adelaide Medical School, Faculty of Health and Medical Sciences, University of Adelaide, Adelaide, SA, Australia.

## Key messages

**Implications for policy makers**

- Viewing knowledge translation (KT) through a complexity lens will challenge the prevailing culture and rules around research funding, incentives, performance and impact metrics of researchers and organizations wishing to implement new knowledge.
- Using complexity and network concepts will help to design and inform KT initiatives prospectively and holistically as well as shaping the retrospective evaluation of specific interventions.
- Policy-makers as well as researchers, clinicians, and other stakeholders need to embrace an interactive, emergent and co-evolutionary role in KT and shape KT, funding and investment strategies accordingly.

**Implications for public**

By thinking about knowledge translation (KT) using complexity and network concepts, the public has more opportunity to be involved in and shape the way new knowledge is created, mobilised and put into everyday practice to help solve challenging and complex problems. Instead of seeing new knowledge as something generated by ‘outside experts,’ individuals and communities can be part of the teams that create and implement solutions to complex problems.
